# Technology for the formation of engineered microvascular network models and their biomedical applications

**DOI:** 10.1186/s40580-024-00416-7

**Published:** 2024-03-02

**Authors:** He Li, Yucheng Shang, Jinfeng Zeng, Michiya Matsusaki

**Affiliations:** 1 Department of Applied Chemistry, Graduate School of Engineering, Osaka University, 2-1 Yamadaoka, Suita, Osaka 565-0871 Japan; 2 Joint Research Laboratory (TOPPAN) for Advanced Cell Regulatory Chemistry, Osaka University, Suita, Osaka Japan

**Keywords:** Microvascular network, Tissue engineering, Biomaterials

## Abstract

Tissue engineering and regenerative medicine have made great progress in recent decades, as the fields of bioengineering, materials science, and stem cell biology have converged, allowing tissue engineers to replicate the structure and function of various levels of the vascular tree. Nonetheless, the lack of a fully functional vascular system to efficiently supply oxygen and nutrients has hindered the clinical application of bioengineered tissues for transplantation. To investigate vascular biology, drug transport, disease progression, and vascularization of engineered tissues for regenerative medicine, we have analyzed different approaches for designing microvascular networks to create models. This review discusses recent advances in the field of microvascular tissue engineering, explores potential future challenges, and offers methodological recommendations.

## Introduction

Tissue engineering is a broad field that uses methods and knowledge from the life sciences, engineering and clinical sciences to construct functional tissues and organs in vitro, providing new tools for basic and applied research [1]. At present, solely in vitro engineered tissues such as skin and cartilage are used in clinical practice [2]. The complexity and density of the blood vessels within the human circulatory system facilitate the efficient transportation of gases and nutrients to cells [3]. The system comprises a hierarchy of vessel type, from the aorta of centimeter size of capillaries measuring just a few microns [4, 5]. However, due to the lack of microvasculature in the tissues or organs constructed in vitro, oxygen, nutrients and waste products cannot be exchanged in the cell-dense structures, and therefore the thickness of these constructs is limited to the diffusion-limiting distance (less than 200 µm) [2, 6].

The microvascular system comprises a dense, highly specialized network of capillaries (10–15 μm) separated by distances of less than 100 μm, providing optimal conditions for the diffusion of gases and the transport of nutrients, metabolites and circulating cells into tissues [7–9]. During embryonic development, the formation of the capillary network is achieved through two distinct processes: vasculogenesis, which involves the formation of new blood vessels, while angiogenesis describes the development of new vessels from pre-existing blood vessels. In the process of vasculogenesis, the inner cells of the aggregate differentiate into hematopoietic precursor cells, while the outer cells differentiate into endothelial cells (ECs). These newly differentiated ECs then connect to form the primary capillary plexus. During angiogenesis, the new vessels sprout, bud, branch, bridge, and remodel, eventually forming a capillary network [5, 7, 10].

The microvascular system plays a crucial role in maintaining healthy tissues. Recent studies suggest that angiogenesis is a key factor in the development of many diseases, including cancer, diabetic retinopathy, autoimmune diseases, cerebral ischemia, cardiovascular disease, and delayed wound healing. Thus, modeling and examining vascular structure and function is crucial for studying the physiology of vascularized tissues and organs in the human body [11–13]. In recent years, there has been growing interest in the generation of networks that model the vasculature and vascularized tissues in vitro, and these models provide new tools for basic and applied research [14–16]. In basic disciplines, microvascular models can be used to examine the effects of various biochemical signals and physical factors on angiogenesis and vasculogenesis [17–20]. An increasing number of potential drugs for the treatment of this disease are being discovered, including several new biochemical pathways, pharmacological targets, and pro- and anti-angiogenic molecules [11]. Furthermore, these microvascular models have extensive usage in drug screening, therapeutic and delivery testing. They are capable of emulating realistic vasoactive responses, enabling broader hypothesis testing, and have the ability to adjust complex experimental conditions while easily controlling single variables [15, 21]. The ultimate goal is to determine whether our advanced in vitro systems can better correlate and predict human responses than experimental animals. In the field of tissue engineering, engineered vascular tissues show promising clinical potential when combined with host organisms [8]. Some highly metabolic solid organs, such as the heart and liver, are still limited by inadequate nutrient and oxygen transport in thick tissues. This question is significant because integrating the microvascular network into solid organs and applying it to repair and replace solid organs in the clinic could have a tremendous impact on human health [22].

**Fig. 1:**
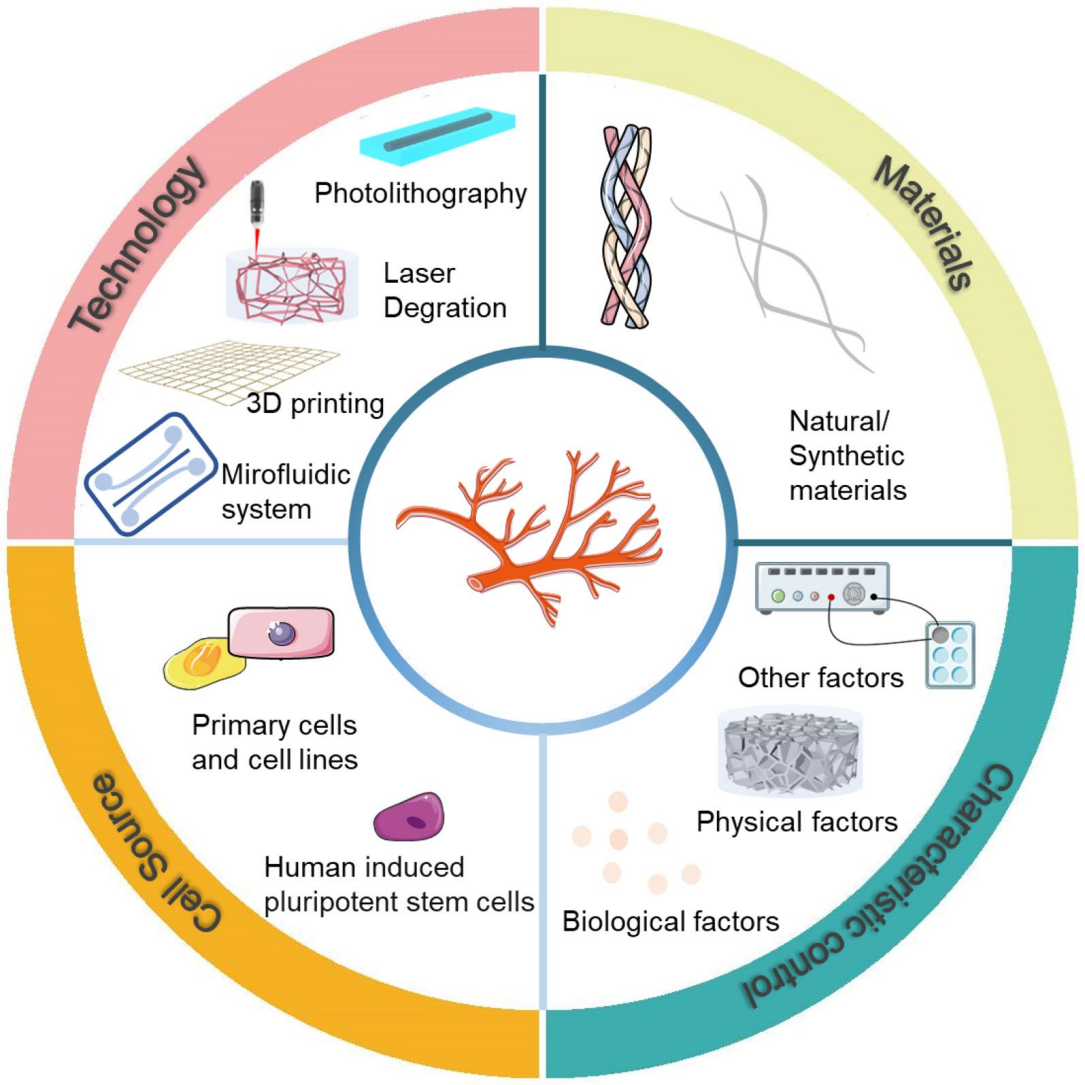
Technology for the formation of engineered microvascular network models (Created at *smart.servier.com*)

Although capillary networks have been extensively utilized in various disciplines and notable advancements have been achieved. Nevertheless, microvascular networks require a different set of design considerations and fabrication techniques due to the intricate geometry and small size of capillaries, compared to those used in large vessel engineering [7, 23]. In recent years, scientists have attempted to create microvascular networks in vitro using different biofabrication techniques and have achieved some progress. This article concentrates on the latest developments in microvascular research and the generation of microvascular-like structures by outlining the latest research that uses various materials, techniques and cellular sources. Finally, challenges and future perspectives in the field are summarized (Fig. 1).

## Technology for microvascular network formation

In general, techniques for engineering vascular networks can be divided into two categories: one is the fabrication of vascular-like channels, and the other is the formation of vascular networks by self-assembly in EC-loaded hydrogels, based on angiogenesis and vasculogenesis [7]. The former refers to the top-down generation of vascular structures by microfabrication methods, with four main options: photolithography, laser degradation, microfluidic systems and three-dimensional (3D) bioprinting. The latter category involves bottom-up self-formation of vascular networks in 3D cell culture [24]. Tissue vascularization based on the top-down approach has no natural biological analogue and is inspired by the surgical approach to tissue reconstruction. It refers to an artificial form of vascularization in which scaffolds are designed to contain permeable channels that can then support the growth of a microvasculature. The underlying motivation is to skip the process of tubulogenesis or lumen formation that is required in the vasculogenic and angiogenic approaches. By avoiding the need for cell migration and invasion, angiogenesis can be accelerated and predefined locations and geometries of vascular networks can be achieved [25]. As it is an unnatural method of vascularization, it relies heavily on patterned biomaterial engineering [26]. The bottom-up approach employs gradients of growth factors and other chemicals in the hydrogel to stimulate EC migration. This process mimics angiogenesis in the body, where new blood vessels sprout from existing ones. However, cell migration and invasion are slow processes, and they do not control the location and geometry of the microvascular network, resulting in poor reproducibility [27, 28].

### Photolithography

Inspired by the principle of lithography, photolithography is a method that uses optical or ultraviolet (UV) light to create specific patterns on a substrate and is widely used in the manufacture of chips and semiconductors [29, 30]. In particular, photolithography is widely used in the fabrication of microvascular networks.

**Fig. 2:**
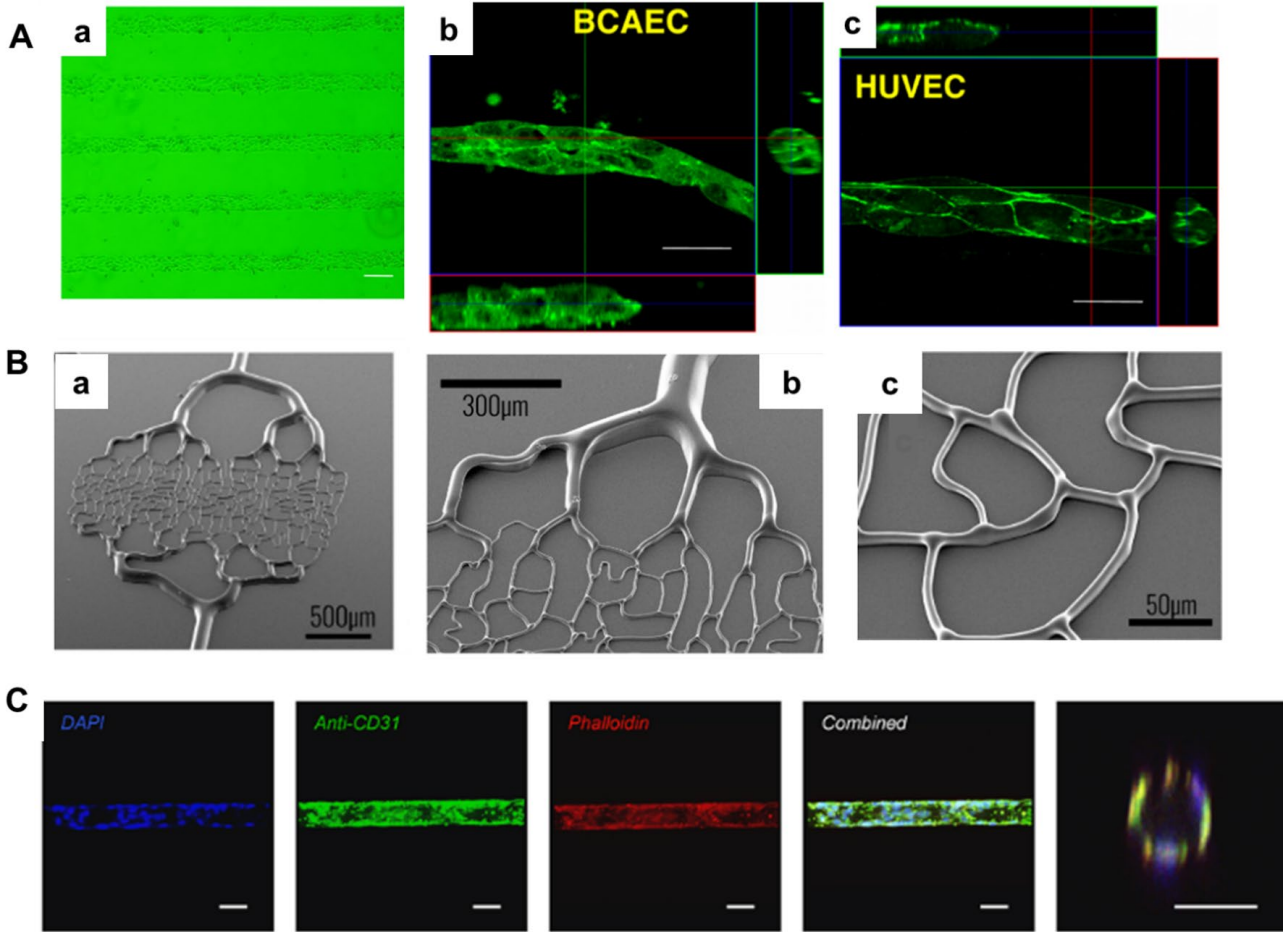
Fabrication of a microvascular network by photolithography. **A** Photolithographic approach to generate cellular micropatterns. **a** Crosslinked chitosan pattern after 180 s of UV exposure. Bar = 100 μm. **b**, **c** Engineered tubular structures of BCAEC and HUVEC scanned by confocal laser-scanning microscopy (CLSM). 3D images showed a lumen within the tubular structures. Bar = 20 μm. (Figure reprinted with permission from Ref. [32]). **B**, **a**–**c** SEM image of the backside lithography technique at different magnifications. We can observe the gradation in height according to the width of the channels. (Figure reprinted with permission from Ref. [36]). **C** Culture of primary human lung microvascular endothelial cells (HLMECs) in the hourglass-shaped channels. Scale bar, 50 µm. (Figure reprinted with permission from Ref. [37])

In 1993, Cokelet et al. constructed straight, perfusable blood vessels on glass plates using a computer-assisted system [31]. However, at this time, the microvascular network only served as a perfusable channel for hemodynamics assays and was not seeded with cells. Soft lithography has been used to generate vascular-like structures in hydrogel materials that allow degradation and remodeling of the surrounding matrix, providing a biologically relevant environment [16]. These biomaterials support cell attachment and infiltration. The adhesion properties of the cells have been used to successfully culture bovine carotid artery endothelial cells (BCAECs) and ECs on patterned substrates to form capillary-like tubes in vitro, which were successfully transplanted in vivo [32] (Fig. 2A). However, these idealized straight capillaries do not accurately reproduce microvascular geometries and cell morphology in vivo. To overcome these limitations, advanced photolithography techniques, including modified geographic information systems [33–35], backside lithography [36] and thermal reflowing of photoresists [37], have been used to fabricate capillary networks with variations in vessel diameter close to the real physiological structure (Fig. 2B, C).

Polydimethylsiloxane (PDMS) is a classic tool for fabricating microvascular networks using photolithography, where researchers pattern photoresists on silicon wafers and fabricate microchannels with specific structures in PDMS. This approach meets the requirements of creating complex blood capillary architectures, but PDMS has a non-selective uptake of oxygen and other hydrophobic molecules and does not provide extracellular cues in biological matrices [38, 39]. Therefore, scientists have not only attempted to create microvascular networks in PDMS but also to use it as a template to create vascular structures in biomaterials [14, 40]. For example, Chaturvedi et al. used PDMS as a channel model and then inoculated a mixture of ECs and collagen. After successful polymerization, the cell cords were removed from the PDMS template and used to guide the formation of patterned capillaries attached to in vivo tissue [42]. Another team used PDMS as a template and introduced microfluidics to fabricate organ model chips for studies of angiogenic sprouting and neo-vessel formation [43]. The role of microfluidics in microvascular formation will be discussed in detail in Sect. 2.4.

### Spatiotemporal material degradation by laser

Conventional photolithography is limited in that it can only generate a 2D planar pattern. Conversely, high-energy pulsed lasers can supply adequate energy (in the megawatt range), to selectively disrupt hydrogel scaffolds for precise material removal in 3D space.

Laser spatial degradation material techniques employing light subtraction strategies have been used to form 3D microchannels in diverse cytocompatible materials [45, 46]. For instance, they have been used to direct the growth of neural synapses in PEG-crosslinked fibrinogen hydrogels [47] and guide the migration of tumor cells in collagen [48]. This method integrates laser degradation of the hydrogel with image-guided laser control using a virtual mask. It allows for dynamic modification of the vessel geometry at any stage and eliminates the need for generating new masters for the iterative design alterations needed for lithography. As a result, it enables programmable 4D control of microvascular networks [49]. Achieving 3D microvascular endothelialization at the capillary scale has proven to be a formidable challenge despite progress in the field. The direct implantation of cells into capillary-sized structures yields lumen occlusion and uneven cell distribution, necessitating alternative methods. Scientists have strived for uniform cell coverage through laser guidance [50] and by designing curved fluidic channels that depend on topographical factors in the angiogenic microenvironment [49].

**Fig. 3:**
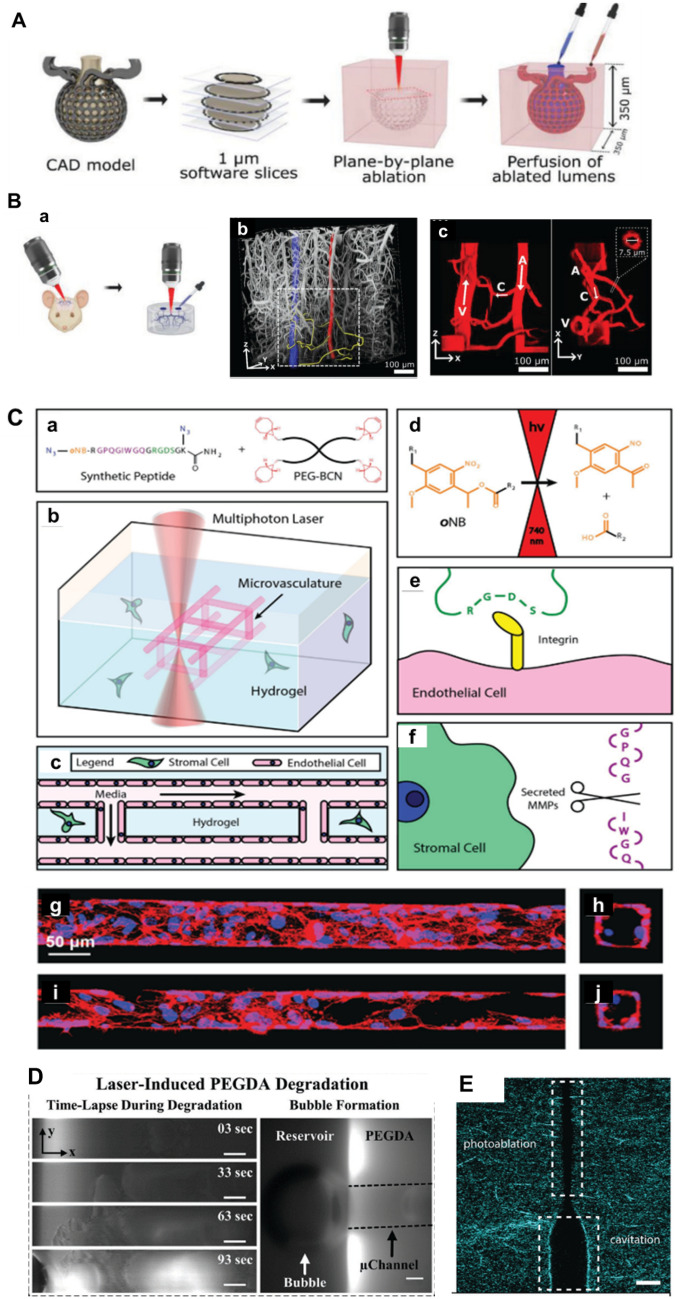
Fabrication of a microvascular network by laser degradation. **A** Schematic of ablation and perfusion process of a human alveolus. **B** Recreation of mouse brain microvasculature. (Figure reprinted with permission from Ref. [52]). **C** Schematic diagram of microvascular fabrication in a multifunctional hydrogel biomaterial. **a**–**f** 3D endothelialized channels generated within photodegradable fluorescent gels. Ten days following microvascular endothelialization with HUVECs, F-actin is shown in red, and nuclei are shown in blue. Endothelialization of **g**, **h** 60 μm × 60 μm and **i**, **j** 45 μm × 45 μm (width × height) channels were obtained. (Figure reprinted with permission from Ref. [49]). **D** Left column: time-lapse images of PEGDA during laser-induced degradation of a 500 × 100 × 100 μm (x, y, z) channel. right column: As microbubbles form, they migrate to the reservoir and coalesce to form a large bubble. (Figure reprinted with permission from Ref. [53]). **E** Laser illumination using 145 nJ pulse energy results in structures in which both photoablation and cavitation-molded sections are present, indicating that this pulse energy is a threshold value at which the transition between the modes of photoablation and cavitation molding occurs. Scale bar, 10 µm. (Figure reprinted with permission from Ref. [54])

Depending on the hydrogel material, laser-induced degradation can occur in three ways [51]. Firstly, photoablation results in extremely localized heating that causes water evaporation and high levels of stress. This leads to the breaking of covalent bonds in the polymer material and the creation of cavities [50, 52] (Fig. 3A, B). Secondly, wavelength-dependent photolysis of chemical bonds occurs in non-specific photosensitive polymer chains [49] (Fig. 3C). Finally, laser-based cavitation molding relies on plasma formation that leads to bubble formation, expansion and cavitation [53] (Fig. 3D). The laser pulse duration, intensity, wavelength, and pore size are influenced by material type and degradation mechanism. Enrico et al. discovered that near the cavitation threshold, laser irradiation results in photoablation or cavitation molding, leading to the formation of various patterns. The cavitation channels take on an almost circular shape, whereas the photoablation structures adopt a teardrop shape, with a pattern height of at least twice the lateral dimension of the channel [54] (Fig. 3E). There is concern over the effects of heat generated by photoablation, as there is the potential for lasers which operate on this principle to negatively impact both the cell and surrounding material. Photopolymer techniques using cytocompatible wavelengths of light [49] and methods based on the cavitation molding mechanism have been used for the creation of 3D microvascular network [54]. This method facilitates the fabrication of high-resolution capillaries with sizes up to 10 μm in diameter. However, the present technique is complex and time consuming, and is essentially a low throughput fabrication method where the fabrication time is related to the size of the tissue. It is valuable for the fabrication of relatively small tissues but inefficient and there remains a need to develop new techniques for obtaining microvascular networks.

### 3D bioprinting

To create larger-sized 3D microvascular networks, much attention has been paid to the use of 3D printing technology. This technology permits the direct fabrication of intricate structures using an extensive range of printing materials [55]. 3D printing can be divided into two main categories. (1) Direct printing, where a material containing cells is deposited on a surface, and (2) indirect printing, where sacrificial materials with printed designs are within larger quantities of material containing cells [56].

#### Direct printing of tissue contents and self-assembly of microvasculature

Direct printing relies on the use of cell-loaded or cell-compatible bioinks to print structures. To maintain the printed structure, rapid stabilization is required, which is achieved by exploiting the high viscosity of the bioinks or by crosslinking the hydrogel in the presence of exogenous factors such as temperature or light to provide adequate mechanical support for the cells [56, 57]. Low-viscosity bioinks require significant technical expertise to process effectively, otherwise they may collapse during extrusion [57]. Conversely, while high-viscosity bioinks are more stable, they can still cause shear stress on cells during printing, which can have a serious impact on the biological activity of those cells [22, 58]. To address these issues, scientists have enhanced the stability of low-viscosity bioinks after cross-linking by utilizing in-situ cross-linking, coaxial extrusion printing, and other techniques. Additionally, they have modified the network structure of polymers [58, 59] or utilized methods like laser printing [60] to improve the rheological properties of high-viscosity bioinks for enhanced cellular bioactivity.

**Fig. 4:**
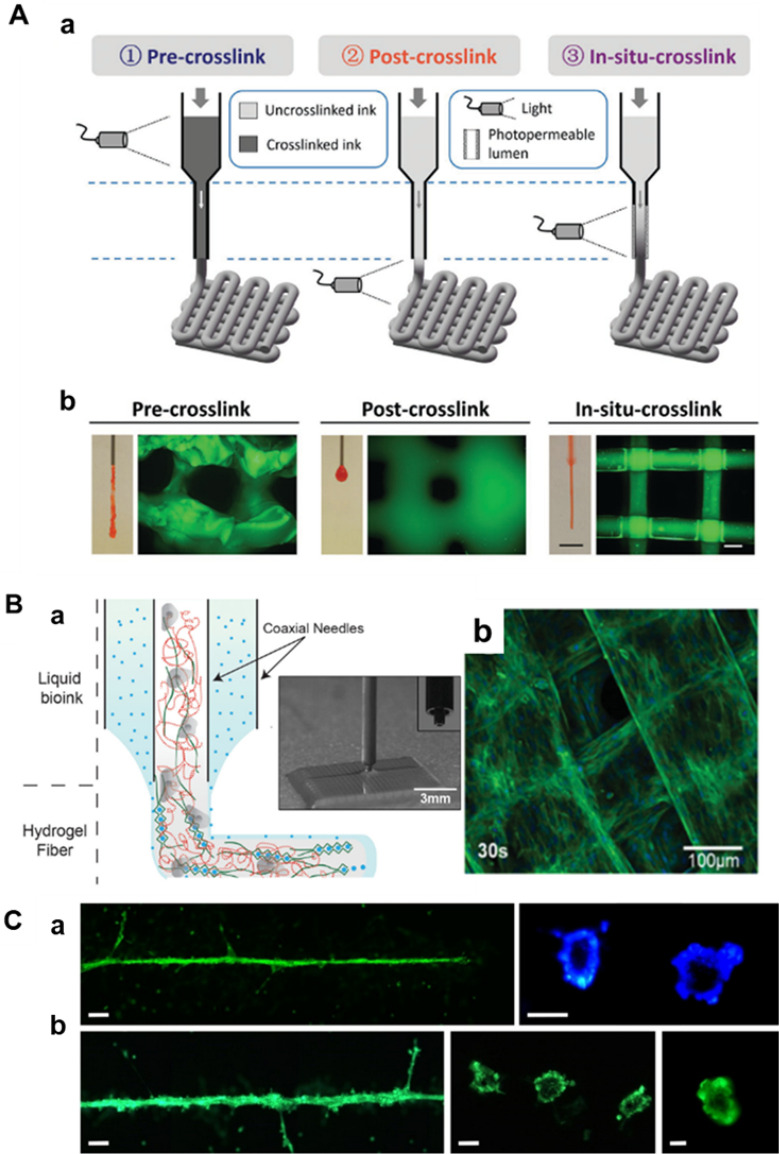
Fabrication of a microvascular network by 3D printing. **A**, **a** Schematic of three different crosslinking strategies for bioprinting photo-crosslinkable inks (e.g., 5 wt% MeHA shown here), where crosslinking occurs before (pre-crosslink), after (post-crosslink), or during (in situ crosslink) extrusion. **b** Representative images of nozzles with extruded material and printed lattice structure. (Figure reprinted with permission from Ref. [61]). **B**, **a** Schematic diagram based on a coaxial bioprinting method. **b** confocal fluorescence image of a bioprinted HUVEC embedded construct under 30 s UV exposure. (Figure reprinted with permission from Ref. [56]). **C** A laser printing method generates capillaries. Green fluorescent endothelial cells. The printed cells formed a tubular structure with a lumen. The applied laser pulse energy was 6 µJ, and the patterns in panel **a** on the left were printed twice in the same place. Scale bars are 200 µm (**a** left and **b** left), 50 µm (**a** right and **b** center), and 10 µm (**c** right). (Figure reprinted with permission from Ref. [64])

In situ crosslink approaches involve the use of photoconductive capillaries to introduce light, which immediately crosslinks the hydrogel prior to deposition of the bioink. The bioink retains a low viscosity before crosslinking, during which time the extrusion and gel crosslinking processes occur concomitantly and expeditiously. This lessens cell damage considerably compared to extrusion occurring pre- or post-crosslink [61] (Fig. 4A). The low viscosity of the bioink also permits the use of thinner nozzles and faster dispensing rates, resulting in higher printing resolution and shorter manufacturing times [62]. In coaxial extrusion bioprinting, bioinks incorporating distinct compositions, like alginate solution and calcium ions, are simultaneously extruded through inner and outer needles. The gel fibers are shaped at the tip of the dispensing system [56]. The low viscosity of these bioinks facilitates the creation of large, porous 3D structures at a high resolution, with a single fiber thickness measuring approximately 100 μm [56, 63] (Fig. 4B). Laser printing technology uses the energy of laser pulses in a jet perpendicular to the surface using a small amount of bioink, which is ultimately deposited to form tissue [64] (Fig. 4C). This process facilitates the development of small capillary networks through high-resolution, high-viscosity, and high cell density bioinks [60, 65]. Dani et al. developed a helical mixing device that produced a homogeneous biological linking after a small number of mixing cycles, resulting in high cell survival [57].

#### Printing sacrificial material to fabricate a tube structure

Due to the difficulties encountered during the early attempts to use direct printing, a method of sacrificial printing has been developed to fabricate microvascular networks. In this method, 3D printed sacrificial materials with microvascular network structures are integrated into bulk hydrogels, resulting in the formation of tubular structures when the sacrificial materials are dissolved [66]. ECs can be integrated in the sacrificial material and attached to the channel wall once it has been removed, or can be added after the channel has been formed. The resolution and capillary dimension of channels formed with sacrificial materials is decreased. The criteria for selecting materials for use in sacrificial templates include rheological properties that meet the requirements of 3D printing technology, mechanical properties that are adequate to maintain the shape and geometry of the printed material during scaffold handling and preparation, and ease of removing the material under non-harsh conditions and without cytotoxic side effects. This results in the formation of hollow channels, which ultimately promote vascularization. The sacrificial materials in use are mainly some thermally responsive bio-sacrificial inks and water-soluble carbohydrate structures [63, 67–69].

A water-soluble carbohydrate material serves as a sacrificial ink to produce 3D microvascular networks, where endothelial cells implant into channel walls to create the eventual vascular bed. This carbohydrate ink confers the required mechanical stiffness to bear its weight, and has broad biocompatibility with the potential to dissolve in a wide range of both natural and synthetic biomaterials [70]. However, these devices typically need to be coated with other substances, such as poly(d-lactic-co-polyethylene glycol) (PDLGA), to slow down the degradation of the leaked ink and avoid damage to cells and the extracellular matrix during the degradation process. Furthermore, this coating is essential for the creation of blood vessels with consistent cell density matrix structures [4]. The novel thermo-responsive material poly(2-cyclopropyl-2-oxazoline) (PcycloPrOx) displays distinctive hydroplasticity where the scaffold gradually becomes plastic once exposed to water. Without additional steps, the material can be rapidly dissolved and removed without compromising the integrity or shape fidelity of the scaffold [71]. Pluronic F127 (PF-127) and gelatin methacrylates possess complementary thermoreversible gelling features, which allow them to serve as sacrificial templates and permanent substrates, respectively, for the creation of endothelialized tubes with a minimum feature size of 30 μm [72].

### Microfluidic systems

Since its emergence in the 1990s, microfluidic technology has been widely used in biology and medicine. Microfluidic methods are characterized by fluid flow control through microchannels with dimensions of tens to hundreds of micrometers [39]. Depending on the mode of angiogenesis, two categories of methods are used, the first of which is the use of endothelial cells that gradually elongate under fluid perfusion and grow against the wall to construct endothelialized microchannels. However, because of limited fabrication technique resolution and the difficulty of perfusing cells into fine channels, it is challenging to produce structurally complex capillary networks with diameters of less than 100 μm [53, 73]. The second process is ECs self-assembly in a suitable microenvironment to form microvascular networks through angiogenesis or vasculogenesis. These blood vessels can be much smaller in size, ranging from approximately 20 to 200 μm in diameter, and they provide a better overview of the capillary network morphology (including branching) as well as the flow and shear stress conditions compared to the solely templated approach [15, 20, 21, 74].

**Fig. 5:**
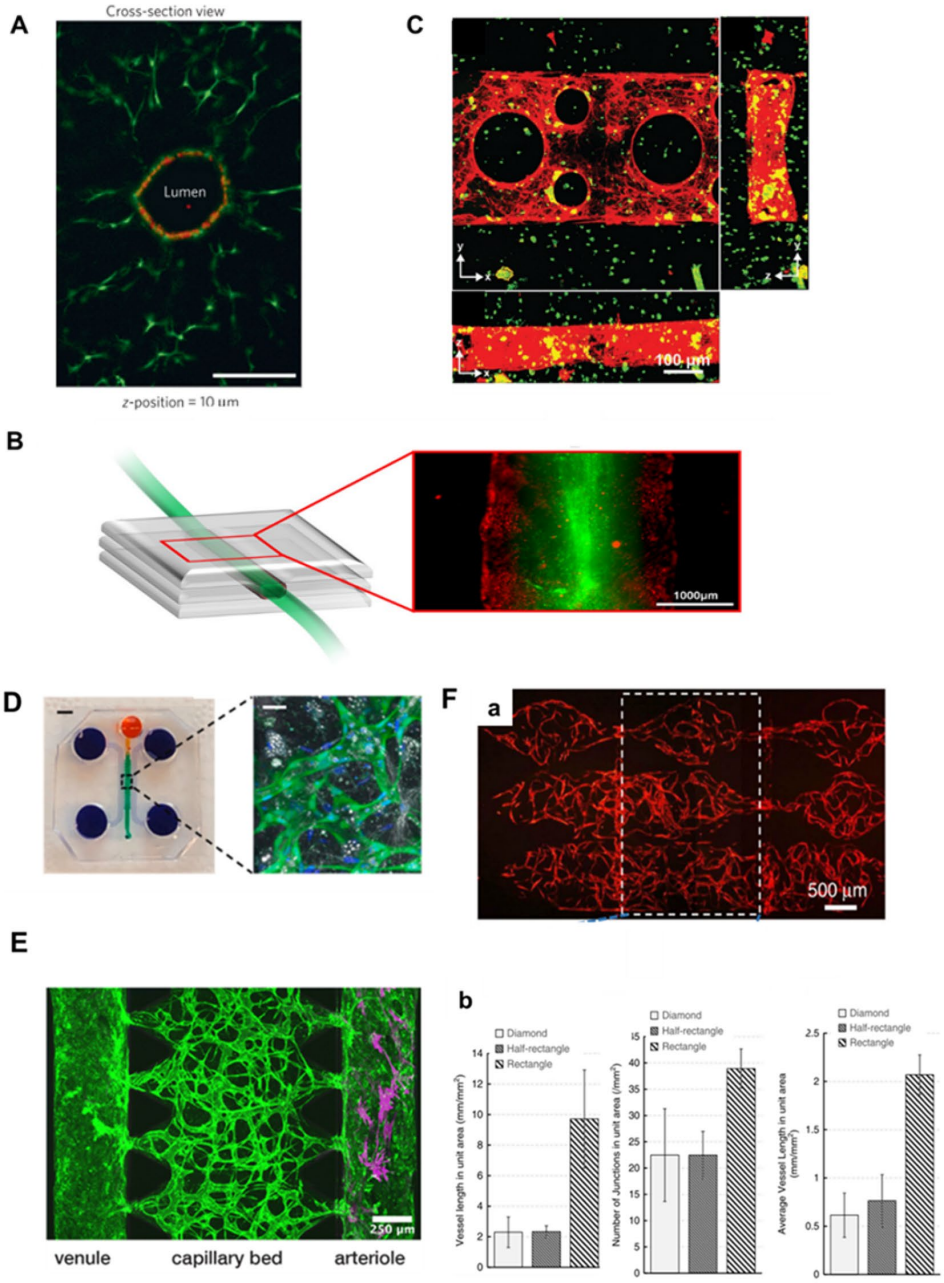
Fabrication of a microvascular network by microfluidic systems. **A** Cross-sectional imaging thickness and Z-position = 10 μm for representative channels (optical channels) after 9 days of microfluidic perfusion culture of endothelial cells in a sacrificial lattice. (Figure reprinted with permission from Ref. [70]). **B**, **a** Schematic diagram of the printed vascular channel construct. **b** Fluorescence image of the printed vascular channel construct by wide-field microscope. HUVECs are visualized in red, beads flow in green. (Figure reprinted with permission from Ref. [69]). **C** Endothelialized channels are readily fabricated in the presence of encapsulated stromal cells. A single-layer channel was generated by photodegradation. Channels were then endothelialized with HUVECs, cultured for 4 days, fixed, and stained for F-actin (red). The sample is viewed (**a**–**c**) as Z-, X-, and Y-direction maximum intensity projections. (Figure reprinted with permission from Ref. [49]). **D** In vitro microvascular network model of the peritoneum. **a** PDMS mold with patterned channels were fabricated using soft lithography and bonded to a glass coverslip. The central gel region (green) contained cells and a fibrin hydrogel. The side channels and reservoirs (purple) as well as the top channel and reservoir (orange) were filled with cell culture medium. Scale bar, 3 mm. **b** A confocal microscopy image of the microvascular networks within the device, in which ECs express GFP, cell nuclei are stained with DAPI (blue), and lipid droplets in Acs are stained with LipidTox (white). Scale bar, 30 μm. (Figure reprinted with permission from Ref. [76]). **E** Create a tricompartmental model of the arteriole-to-capillary-to-venule microvasculature. Capillaries (middle) modeled by perfusable MVNs made from endothelial cells (EC, green) and fibroblasts (FB) in fibrin gel, venule (left) modeled by collagen channel with EC monolayer, arteriole (right) modeled by collagen channel with smooth muscle cells (SMC, magenta); the scale bar is 250 μm. (Figure reprinted with permission from Ref. [77]) **F** The modular microfluidic system combines two PDMS layers. The different morphological properties of the capillaries generated using diamond-, half-rectangle and rectangle-shaped chambers were analyzed. The rectangle-shaped tissue chambers generated the largest capillaries. (Figure reprinted with permission from Ref. [79])

Templating-based techniques can construct microfluidic microvascular models where the geometry and size of the final microvasculature are determined primarily by the employed microchannel template. Using sacrificial printing, Miller et al. have recently presented a method of constructing dynamically perfused vascular channels containing intraluminal EC liners in a hydrogel. The capillary-like 3D vascular networks that were created with microchannels were obtained after removing the lattice embedded in the cell-mixed EC with a cell culture medium [70] (Fig. 5A). This study has a key feature, which is the involvement of cells in the fabrication process, which could have potentially harmful effect. The vascular network within a PDMS microfluidic chip using a sodium alginate lattice as a sacrificial template is different from other previously reported template-based methods. The main feature of the method is that the microvascular network can be prepared prior to the embedding of the constructs in the cell-mixing hydrogel, which completely separates the template removal and cell seeding steps. This prevents any potential harm to the cells loaded in the bulk hydrogel during the fabrication of microchannels, thus maintaining their integrity [69, 75] (Fig. 5B). Despite the limitations of 3D printing and lithography resolution, laser degradation techniques enable the creation of microchannels with high resolution and capillary-sized dimension [22]. Human umbilical vein endothelial cells (HUVECs) are introduced into these microchannels via microfluidics and attach to the lumens, forming a well-defined lumen that covers the wall of the photodegraded channel, which is endothelialized up to a lumen diameter of 45 μm [49]. To enhance cell seeding efficiency, and avoid cell accumulation and clogging in small channels, selectively curved interconnected channels are often used to facilitate microfluidic flow, highlighting the potential of channel microtopography [49, 53] (Fig. 5C).

As an illustration of organ-specific vascular microfluidics, which relies on self-assembly to form capillaries, Ibrahim et al. cultured ECs and adipocytes in the central channel of a microfluidic chip device. Following the creation of the microvascular network, mesothelial and tumor cells were cultured within the microchannel, which allowed for successful crosstalk between the tumor cells and the perfusable microvasculature. This peritoneal tumor model can be used to study the role of mesothelial, endothelial and adipocyte function in tumor metastasis [76] (Fig. 5D). Due to variations in size, flow rate, and endothelial barrier type of microvascular networks in different tissues and organs, the development of tissue- and organ-specific perfusable vascular networks requires flexibility in design [9, 39, 78]. The complex in vitro system must consider the cellular composition and biophysical microenvironment experienced by various blood vessels. Scientists have attempted to address these issues by designing multi-chamber microfluidic systems. Modular microfluidic devices have been used for the separation of tissue chambers and medium channels. The medium flows vertically into numerous tissue chambers while the parameters of both channel layers can be adjusted. By modifying the interstitial flow pattern, microvascular networks of varying lengths and widths can be effectively culture [79] (Fig. 5F). As Chesnais et al. noted, flow has a marked impact on capillary development, cell migration, stromal cell remodeling of the extracellular matrix, and bud formation. A microfluidic drive system for microfluidic chips has been developed that can be easily assembled and mounted in cell culture chambers. Computational fluid dynamics analyses have indicated that the system is capable of achieving a stable and turbulence-free flow rate (10–1000 μl min^−1^), achieving the desired bionic flow rate and direction [80, 81]. Chen et al. designed a three-chamber microfluidic chip, which was seeded with ECs in a collagen matrix. They successfully constructed a microvascular network that contained complete small arteries, capillaries, and small vein [77] (Fig. 5E). Compared with some single-chamber microfluidic chips, the 3D structure and physiological functions of human microvasculature are more completely represented. The primary advantages of these hierarchical models are the simultaneous analysis and direct comparison of vascular chambers, the ability to adapt to complex experimental conditions, and the ability to connect multiple organ-on-a-chip models on the same platform, enabling flexibility and scalability of in vitro microvascular networks (Table 1). These engineered vascular engineering models excel at simulating the intricate physiological microenvironment present in living systems. At the same time, these microvascular systems can often be utilized by combining them with a variety of quantifiable and scalable assays, such as macromolecular permeability, image-based screening, Luminex, and qPCR [15]. Thus, these models offer convenience and reliability for studying tissues, including researching the molecular mechanisms of angiogenesis [14, 16, 43]. They are widely used in applications such as testing therapeutic delivery [79, 82], modeling cancer metastasis [83] and analyzing immune cell behavior [15].

**Table 1:** Techniques for microvascular network formation

Technology	Size (μm)	Advantages	Limitations	Ref.
Photolithography	5–130	Convenient, cost-effective	Unchangeable	[37, 41, 44]
Laser degradation	10	Programmable, user-defined	Low-throughput, time-consuming	[49, 50]
Direct printing	100	User-defined	Cell damage	[61, 62]
Sacrificial bioprinting	150–500	User-defined, High speed	Low resolution	[67, 70]
Microfluidic systems	5–200	To reproduce vascular related physiological or pathological processes	Lack of control over vascular geometry, low repeatability	[53, 80]

## Materials for microvascular network formation

Angiogenesis and vasculogenesis rely on the migration and growth of cells. The main components of the extracellular matrix (ECM) are collagen fibers, fibronectin, hyaluronic acid, vitronectin and fibronectin which provide a favorable environment for cells to proliferate and migrate. Hydrogels, because of their structural and biochemical similarity to extracellular matrices, are the materials of choice for creating microvascular networks in vitro [84]. Biocompatibility is a key consideration in the selection of hydrogels, and it is important to ensure that the chosen hydrogel does not have a detrimental effect on the biological system [22]. These materials fall into two categories: natural polymers and synthetic polymers, and the biological properties of the various materials are summarized in Table 2.

**Table 2:** Properties of various biomaterials for microvascular network formation

Material	Material type	Properties	Ref.
Collagen	Natural	High mechanical strength, can be polymerized with different materials	[86, 88, 89]
Fibrin	Natural	Requires thrombin activation; Readily biodegradation properties; Poor mechanical strength; High biocompatibility	[87, 91]
Matrigel	Natural	The composition is unidentified, however, it offers an adequate environment for the proliferation of cells	[89, 100, 102]
Gelatin	Natural	Promotes and maintains cell viability; Biodegradable	[104]
Alginate	Natural	Controlled biodegradation, low viscosity, and crosslinking with Ca^2+^ to form gel fibers that are optimal materials for bioprinting	[56, 107]
GelMA	Synthetic	Provides a biocompatible and hydrophilic environment for cells. The mechanical strength can be adjusted as required	[56, 111]
PEG/ PEGDA	Synthetic	It is non-biodegradable, exhibits weak mechanical properties, lacks suitability for cell culture, and necessitates the use of binding peptides or degrading enzymes	[53, 113]
PLA	Synthetic	Biocompatibility, degradability is appropriate, high hydrophobicity, needs to be combined with hydrophilic materials	[115, 116]

### Natural polymers

Natural polymers such as collagen, fibrin and Matrigel are the major components of the ECM within most tissues. They provide cell adhesion sites, are remodeled by cells and degraded to release pro-angiogenic morphogens, such as growth factors and ECM fragments, which promote angiogenesis and vasculogenesis [85].

Collagen is the primary structure that creates the supportive framework of the vascular ECM [86]. The tropocollagen molecule, an active terminal polypeptide that requires no further activation, is typically used to generate hydrogels [87]. Its biodegradability in vivo occurs mainly through collagenase and histone enzymes, leading to high retention periods of collagen hydrogels in vivo, usually for weeks [87]. The high mechanical strength of collagen hydrogels stems from the combination of collagen fibers organized in a staggered and overlapping manner, leading to fiber synergism and, overall, the high tensile strength of higher order structures [88]. Collagen has a crucial function in shaping ECs and arranging them into a 3D vascular-like network. Sufficient vascularization has been reported to be in the collagen concentration range of 1.5–6 mg/ml, with a storage moduli in this range of 385–510 Pa [89]. The microstructure of collagen in the ECM determines the structure of the vascular network, and regulating collagen deposition and the magnitude of traction forces exerted by cells can manipulate the formation and alignment of vascular-like structure [84].

Fibrin gels possess the ability to advance cell migration, proliferation, and matrix synthesis by binding transforming growth factor β and platelet-derived growth factor [90]. Fibrin hydrogels are produced from inactivated fibrinogen molecules and therefore require an additional activation step by thrombin [87]. Fibrin is typically incorporated within scaffolds due to its superior cell adhesion property, which is linked with a greater affinity of integrins towards it. Additionally, it can modify stiffness parameters by modulating the blend of thrombin, fibrinogen and factor XIII [91, 92]. In vivo, plasmin is constantly produced in a cyclic, inactive form that breaks down fibrin. Hence, fibrin gels are transient biomaterials that are typically biodegraded within a few days [87]. However, fibrin gels possess unsatisfactory mechanical properties and are vulnerable to cell-mediated shrinkage and mechanical strain following in vivo implantation. Scientists have sought to enhance tissue strength and cellular activity by bonding fibrin to biomaterials such as collagen using standard crosslinking agents such as glutaraldehyde, the natural extract genipin and glycosaminoglycan resulting in multi-component collagen scaffolds with higher vascular volumes than tissue constructs using fibrin gels alone [93–96]. Researchers have incorporated additional cells (e.g. fibroblasts) into fibrin gels to increase the mechanical robustness and stability of the material, as cellular components can contribute to ECM deposition and improve structural integrity [97–99].

Matrigel, an ECM complex obtained from mouse tumor tissue comprising laminin-1, collagen IV and other matrix fragment proteins, has been demonstrated to enhance angiogenesis, protease activity, tumor growth and metastasis [100]. It has a broad range of beneficial applications in tissue engineering. Due to its exceptional pro-angiogenic properties, Matrigel has been established as a standard substrate for EC tube formation assays and in vivo angiogenesis assays. Additionally, it can be used as a complement to other materials while consistently maintaining its pro-angiogenic effects [89, 101]. Schumann et al. demonstrated that pre-culturing mesenchymal stem cells (MSCs) in Matrigel is a promising approach for achieving rapid growth of microvasculature in tissue-engineered constructs [101]. However, Matrigel contains numerous undefined protein components so its use in microvascular network formation is limited due to factors such as batch-to-batch compositional variability, variability between mechanical and biochemical properties, and immunogenicity induced by animal-derived matrices [89, 102, 103].

Gelatin is a protein derived from collagen through hydrolysis and simulates natural tissues, giving it certain properties that promote angiogenesis. Its thermos reversible nature endows gelatin with physical gel properties at low temperatures (20–30 °C) which melt at physiological temperatures [104]. Gelatin displays good cell retention properties and appropriate matrix viscosity, and is thus a key component in producing extrusion printed bioinks and sacrificial printing [68, 69, 105].

Alginate, a hydrophilic polysaccharide, exhibits pro-angiogenic properties when combined with other stable, gel-forming, and cell-responsive biopolymers, creating an ideal microenvironment for embedded cells [106]. The solubility of alginate is easily achieved in the absence of calcium ions, has no cell binding sites and offers controlled biodegradability [107, 108]. Alginate-chitosan hybrid microcapsule scaffolds guide the alignment of HUVECs and provide support for the formation of vascular-like networks [109]. Calcium ions are often required to crosslink alginate in 3D printing for hydrogel microfiber formation. The concentration of CaCl_2_ solution should be strictly controlled to minimize cytotoxicity caused by calcium ions [56, 110].

### Synthetic polymers

Synthetic polymers exhibit remarkable versatility because their mechanical properties can be easily controlled, making them a flexible and customizable means of constructing scaffolds. The processing of scaffolds based on synthetic polymers can be carried out under mild conditions and at a reasonable cost. However, these scaffolds lack key biological cues that influence cell adhesion for growth.

Gelatin methacrylate (GelMA) is a modified, denatured collagen with photopolymerizable methacrylate (MA) groups at the amino-terminal end of gelatin to form hydrogels that can be covalently crosslinked under UV light [111]. GelMA has properties that are representative of both natural and synthetic biomaterials. Gelatin possesses integrin-binding motifs and matrix metalloproteinase-sensitive fragments, allowing for cell-responsive properties that include appropriate cell adhesion sites and hydrolytic protein degradation [111]. The shear yield stress and Young's modulus of the hydrogel system can be altered by changing the concentration, degree of methacrylation and temperature [112]. Enhanced compression modulus may be achieved with increased levels of methacrylation and concentration of hydrogel. GelMA hydrogels with low concentration (< 5% w/v GelMA) and low degree of acrylamide modification exhibited spontaneous organization of the encapsulated cells, including human mesenchymal stem cells (hMSCs) and ECs. This is in contrast to GelMA hydrogels with a high concentration (> 10% w/v GelMA) and/or high degree of acrylamide modification [56].

Polyethylene glycol (PEG) is a hydrophilic polymer that is commonly used in biocompatible tissue engineering scaffolds. However, PEG is non-biodegradable and has inferior mechanical properties. Despite this, PEG degradation can be induced by enzymes and hydrolysis [112, 113]. More recently, modular and multi-functional hydrogels based on PEG have been developed for specific applications. The materials are covalently and physically crosslinked under light irradiation in the presence of photoinitiators in some synthetic materials based on PEG containing photocrosslinking groups, including poly (ethylene glycol) diacrylate (PEGDA) [50, 53]. The choice and concentration of the photoinitiator can markedly affect the cytocompatibility and cell viability of the substance [114]. PEGDA and other similar synthetic polymers are photoactive but lack the bioactive signals inherent in natural ECM. Consequently, they are less proficient at supporting the growth of live cells in various tissues, including engineered vascular networks and their adjacent tissues. To overcome this limitation and endow additional ECM properties, researchers have endeavored to modify photopolymer materials by linking peptides and degradable enzymes that promote cell adherence and proliferation [49].

Polylactic acid (PLA) is a polymer with a reliable biodegradable nature and cytocompatibility and mechanical stability [115]. However, its linear structure and hydrophobic properties lead to poor mechanical stiffness and degradation products that limit tissue regeneration, making it an unsuitable material for scaffolding [116]. Therefore, PLA is often combined with other materials, such as PLGA, to be successfully fabricated as a scaffold material [117].

## Cell source for blood capillary formation

Natural capillaries consist of ECs surrounded by a basal layer and a single layer of pericytes. A thin layer of connective tissue that is contiguous with the connective tissue of the surrounding tissue makes up the outermost layer of the capillary [118]. The vascular endothelial structure also shows heterogeneity at the ultrastructural level, due to the functional heterogeneity of organs [119]. For instance, in organs like the kidney and liver, the endothelium is discontinuous, permitting fluid and molecular exchange [120]. In the cerebral vasculature, the blood–brain barrier is made up of tightly packed ECs that are largely surrounded by pericytes, restricting paracellular transportation of specific transport proteins [121]. The characteristics of the various cell types are summarized in Table 3.

**Table 3:** Advantages and disadvantages of cell types used for microvascular network formation

Cell type	Advantages	Disadvantages	Ref.
Primary cells	Easy to obtain, closest to the body’s cells	Acquiring and isolating primary cells is a costly and time-consuming process, complicated by the heterogeneity and inter-donor variability	[122]
Cell lines	Standardization, scale, low cost	The genome of the cell has been modified, resulting in the absence of certain traits present in the primary cell	[119, 128]
iPSCs	Provides tissue specific cell types	Multiple differentiation is necessary to acquire target cells	[42, 132]

### Primary cells

The development of the vasculature is a complex process, with functional specialization occurring early in vascular development, and vascular cells such as ECs and pericytes have heterogeneous transcriptional profiles in different tissues and within the same tissue. Therefore, engineering tissue-specific blood vessels necessitates the use of such specialized cellular components [122]. Primary cells (such as HUVECs) are extensively used in bioengineering to construct vascular systems at a microscopic level owing to their easy accessibility and their close resemblance to real cells in vivo.

Although ECs, which make up the innermost layer of blood vessels, can naturally form initial networks, the absence of pericyte recruitment causes these structures to rapidly deteriorate [33, 123]. Pericytes have an important role to play in the regulation of vascular blood flow, regulation of the internal diameter of the vasculature, and vascular stabilization [124, 125]. Although the communication between pericytes and ECs remains to be elucidated, studies in this area have established that their interaction during angiogenesis is regulated in part by Ang-1/Tie2, TGF-β1 and PDGFB/PDGFR-β signaling [124]. Whisler et al. observed that co-cultivation of HUVECs with fibroblasts led to robust EC sprouting, resulting in neovascularization in just 4–5 days. The developing vascular network retained a constant shape and perfusion capability [126]. In addition to providing growth support, stromal cells have been found to surround endothelial cells and exhibit pericyte-like behavior. Fibroblasts influence vascular network formation and vascular stabilization processes through direct paracrine signaling and secretion of molecules that regulate the ECM [127]. However, due to the time and cost involved in obtaining and isolating primary cells, as well as the variability of individual donors, scientists have begun to experiment with culturing phenotypically stable cell lines.

### Cell lines

In order to standardize and scale the construction of microvascular models, scientists have attempted to establish immortalized cell lines to complete the fabrication of vascular networks. Although the establishment of permanent cell lines ensures the stability of the cellular phenotype and reduces costs, the unlimited proliferation of cells often leads to changes in the cellular genome. In other words, the cells no longer resemble their parent cells [119, 126].

This has led researchers to seek improvements in cell line cloning techniques to achieve a genomic phenotype almost identical to that of the progenitor cells. Human pulmonary microvascular endothelial cells (HPMEC-ST1.6R) that have been cloned with success have been shown to be non-tumorigenic in nude mice while requiring serum for in vitro proliferation. This valuable resource of a cloned MEC cell line, HPMEC-ST1.6R, serves for the study of microvascular endothelium for physiological and pathological conditions [119]. However, some common vascular ECs lines, such as the HUVECs cell line, often require processing and pre-validation to identify donor sources with similar functions when used for in vitro angiogenesis standardization screening and angiogenesis experiments [128].

### Stem cells

While many engineering platforms have managed to build microvascular networks using primary endothelial cells and engineered immortalized cell lines, the heterogeneity of primary human endothelial cells and the de-differentiated nature of immortalized cells cultured in vitro cause the absence of certain specific physiological characteristics. This limitation markedly hinders the standardization and scaling of engineered blood vessels. To meet the requirements of emerging tissue modeling applications that depend on standardization and quantification, it is crucial to construct robust models of the vascular network [129]. Previous studies have used non-brain-specific endothelial cells, such as HUVECs or dermal microvascular endothelial cells, to develop blood–brain barrier models or cerebral microvascular models for drug screening purposes. However, these cells lack the crucial features of brain microvascular endothelial cells (BMECs). High trans-endothelial electrical resistance limits paracellular permeability and exocytosis activity, making these models unsuitable for studying the characteristic features of vascular networks [121, 130, 131].

Human pluripotent stem cells (hPSCs), which consist of human embryonic stem cells (hESCs) and human induced pluripotent stem cells (hiPSCs), have been extensively used to study somatic cell types due to the ability to obtain young vascular cells from the same source [132]. The process of stem cell differentiation and tissue morphogenesis largely depends on well-coordinated spatiotemporal signaling mechanisms and tissue architecture [133]. Several research groups have endeavored to create induced pluripotent stem cell-derived endothelial cells (iPSC-ECs) that express CD 31 and CD 144 antigens [132]. In a study by David et al., iPS-ECs were inserted into a fibrin gel within a microfluidic device and co-cultured with normal human fibroblasts. These fibroblasts were then arranged into a 3D network of interconnected capillaries. Ultimately, the successful assembly of a 3D network of interconnected capillaries was achieved [129]. iPSC-ECs alone are not sufficient to form robust vascular networks. Kusuma et al. attempted to induce co-differentiation of hPSCs into early vascular cells through a clinically relevant strategy applicable to multiple hPSC differentiation. These early vascular cells can mature into ECs and pericytes and self-organize in engineered matrices to form microvascular networks and bind to the host vascular system [132]. The rapid formation of a robust vascular network has been observed in immunodeficient mice upon co-implantation of ECs with pericyte progenitors, in contrast to single-source iPSC-ECs. hPSC pericytes were essential for the neogenesis of these vessels which exhibited the presence of pericytes surrounding the engineered blood vessels [134]. Distinguishing perivascular cell types is challenging for hPSCs cell differentiation because of overlapping marker expression and comparable functions [135, 136]. The genes related to endothelial cells such as PECAM1, VE-cad, and GATA-2 are strongly expressed after the formation and differentiation of ECs. A recent investigation conducted by Cho’s team discovered that iPSC-ECs efficiently reconstructed ischemic retina in comparison to human primary endothelial cells. This implies that iPSC-ECs exhibit a stronger CXCL12/CXCR4 chemotactic relationship [137]. Relying on potentially autologous, highly productive and unlimited cell sources of pluripotent stem cells in engineered microscale vascular systems may be the most effective way to create in vitro models and vascular tissues.

## Characteristic control of microvascular networks

### Biological factors

The extracellular matrix stores and releases growth factors in response to physiological and pathological stimuli. These factors bind to cellular receptors, activating downstream signaling pathways that angiogenesis and vasculogenesis [138, 139]. Several methods have been developed to replicate this process by incorporating growth factors into biomaterials to manage vascular growth and development in vitro. The signaling proteins that predominantly affect angiogenesis are vascular endothelial growth factor (VEGF), acidic fibroblast growth factor (aFGF), and basic fibroblast growth factor (bFGF) [140]. Son et al. mapped the topography of the growth factor gradient using growth factor-secreting cells. The endothelial cells along the direction of the growth factor gradient to generate capillaries [141].

Although the formation of capillary-like structures is typically induced by the addition of growth factors, such factors are typically costly, released quickly, and have a short half-life [142]. To address this question, researchers have employed slow-release approaches and gene editing techniques. Growth factors may be covalently attached to the matrix or loaded into microparticles to enable sustained delivery [143]. Munarin et al. conducted sulfate and heparin fraction covalent coupling with alginate, mimicking specific heparin-binding growth factor (including VEGF and bFGF) binding to extracellular matrix components such as laminin, fibronectin, and fibrinogen. As a local depot for delivering vascular endothelial growth factor (VEGF), VEGF can be released continuously for over 14 days [144].

Simultaneously, it has been observed that cells operating in angiogenesis, such as endothelial and pericytes, secrete cytokines that encourage angiogenesis [145]. A group of N_2_-polarized neutrophils have been identified with anti-inflammatory properties, which effectively prevent the recruitment of inflammatory cells. They also maintain the survival of exogenous endothelial cells and promote angiogenesis through vascular anastomosis and maturation [146]. Xu et al. used gene engineering to express collagen-binding domain fusion factors and created matrix materials enriched with VEGFA and bFGF to encourage angiogenesis [147]. In addition, the delivery of genes coding for angiogenic growth factors to cells in microenvironments through cell-carrying scaffolds of transfected cells induced a controlled release of angiogenic proteins and promoted angiogenesis. This controlled release of proteins promotes angiogenesis while simultaneously providing structural support for neo-matrix deposition. This approach provides a novel, selective, and alternative way to influence vascularization during tissue regeneration [142, 148].

### Physical factors

In general, even small changes in the 3D microenvironment, such as scaffold stiffness, can affect the behavioral expression of cells [149]. A study investigated the impact of hydrogel mechanical properties on capillary self-assembly and found that softer matrices, which were more conducive to EC migration, led to longer lengths and more branches within the capillary networks. In contrast, the proliferation and migration of ECs were limited in stiffer matrices, which resulted in reduced tube-forming capacity [150]. It is important to note that various microvascular fabrication techniques necessitate distinctive material matrices. For instance, when utilizing microfabrication techniques such as photolithography that necessitate retention of morphological characteristics even after demolding, harder materials like PDMS are commonly employed [40]. 3D printing techniques require the material to maintain high fluidity at the start and then quickly become stiffer for sufficient mechanical support of the cells [23]. The stiffness of the matrix can be modulated through variation in the polymer or crosslinking agent, or by increasing the enzyme cleavage sites [151]. However, these changes also affect other factors such as matrix density and pore size, which limits its role in angiogenesis and vasculogenesis.

The stiffness of the matrix plays a crucial role in regulating microvascular growth direction. Due to the boundary constraints of the hydrogel, it cannot be separated from the scaffold surface, and the stiffness gradient near the scaffold surface is steeper [152]. Under these conditions, when cells contract and impose constraints on the hydrogel, the boundary restricts gel deformation, leading to increased cellular tension. Culturing hydrogels containing endothelial cells within rectangular matrix boundaries aligns the cells along the long axis, producing a stronger effective stiffness, ultimately resulting in oriented microvascular [153, 154]. The stiffness of the matrix also affects the ability of microvascular generating buds to signal orientation awareness, with capillaries displaying a greater tendency to align with the VEGF gradient in a stiffer ECM [145, 146]. Despite soft matrices supporting angiogenesis, the directional inclination of angiogenesis is stronger in hard matrices. Chiou et al. conducted a comparison between the length of capillaries grown on hydroxyapatite scaffolds with pore sizes of 450 μm and 250 μm. Results indicated longer vessels and increased branching within the 250 μm pore at 14 days [152].

Physiological shear stress is typically associated with sustained unidirectional laminar blood flow through capillaries. Under static conditions, cells exhibit a cobblestone-like shape. However, shear stress can induce directional alignment and elongation of endothelial cells by activating intracellular signaling pathways and transcription factors, while also regulating gene expression, enhancing cell–cell junctions and cell–matrix adhesion, and decreasing permeability [155–158]. Helms et al. investigated the influence of shear stress on vascular growth directionality. Shear stress induced physiological alignment of capillaries parallel to the longitudinal vessel axis, resulting in 57.2 ± 5.2% of cells aligned within 5° of the main blood vessel axis. In contrast, cells incubated without stimulation exhibited a randomly oriented tube formation [3]. Liu et al. used shear stress generated by the dispensing process to align collagen microfibrils, which combined with integrin-induced adhesion to guide cell alignment. Consequently, directional growth of capillaries was achieved [159]. Furthermore, various types of ECs exposed to the same orientation demonstrated analogous responses under high shear stress. However, microvascular cells demonstrated a more sensitive reaction to lower stimuli alterations in orientation, compared to ECs from vessels with larger diameters [157]. Shear stress triggers a cascade of autocrine and paracrine signaling events between ECs and other cells, promotes fibroblast proliferation, and stimulates the release of bFGF [145, 155]. Cells entered the scaffolds more under flow conditions than under static conditions, successfully overcoming the problem of cell adhesion due to the complex scaffold structure, narrow pore sizes, and materials that resulted in uneven cell seeding and insufficient initial cell seeding [157]. This cell seeding method, using microfluidics as the main method, helps to improve seeding efficiency and offers the possibility of uniform cell distribution throughout the matrix [50]. Recently developed perfusion bioreactors can apply flow-induced shear stress to vascularized 3D tissues. Direct flow conditions have been shown to induce microvascular formation and increase vessel length, density, and maturation [80, 160].

### Other factors

Chemical factors, such as oxygen levels, have been shown to influence angiogenesis in vivo. While it is widely accepted that activation of hypoxia-inducible factor-1a (HIF-1a) under low oxygen conditions induces erythropoiesis and angiogenesis, normal oxygen levels promote the growth and proliferation of ECs. Therefore, the use of O_2_-producing hydrogels is expected to maintain the survival and function of peripheral or infiltrating cells prior to local vascular reconstruction, which is essential for the tissue regeneration process [161, 162]. Gao et al. introduced oxygen-producing calcium peroxide (CaO_2_) into Ca^2+^ crosslinked alginate hydrogels. The addition of CaO_2_ provides dynamic crosslinking and increases local oxygen concentration. As CaO_2_ decomposes, local oxygen reduces intracellular reactive oxygen species (ROS) and HIF-1a levels, improves cell viability, promotes cell migration and angiogenesis, and provides a better microenvironment for cell growth and potential tissue regeneration [163]. Tran et al. found no significant difference in cerebral microvascular germination between normoxia and 1% oxygen. However, an external oxygen gradient significantly increased neovascularization sprouting, leading to angiogenesis [164].

In recent years, several studies have shown that electric fields (EF) can promote angiogenesis by stimulating the production of angiogenic factors [165]. However, most studies exploring the potential of electrical stimulation to promote angiogenesis are preliminary. Early animal studies showed that electrical stimulation induced an increase in the density of muscle capillaries [166]. Studies conducted in 2D cell culture have shown that stimulation with an EF activates downstream Rho-ROCK and PI3K-Akt signaling through vascular endothelial growth factor receptor (VEGFR), leading to cytoskeletal reorganization. This results in significant cell elongation, directed and oriented migration, and induces an angiogenic response [167]. EF induces directed migration and significantly increases cell migration rates in human microvascular endothelial cells (HMEC) and HUVEC [168]. Chen et al. used in vitro 3D culture to examine the effects of EF over time on the ability of endothelial cells to form tubular networks. The study found that EF stimulation impacts the organization of tubular structures, which is linked to the activation of VEGFR-2-mediated signaling pathways by VEGF that regulate endothelial cell migration and proliferation. These findings are consistent with those in a 2D cell model [169]. Lu et al. also explored the use of electrical stimulation in microvascular tissues. The study found that both physiological-intensity electrical stimulation and high-intensity electrical stimulation significantly increased the expression of relevant genes, such as CD31, CD144, and CD34 in HUVECs. Immunofluorescence staining showed the morphological changes and the emergence of capillary-like structures in HUVECs, achieving ECs vascularization of engineered cardiac tissues [170]. While electrical stimulation has been utilized in microvascular tissue engineering, it is necessary to further investigate the optimal force intensity of such electromechanical stimulation and its target signaling pathways.

## Conclusions

This article examines recent developments in microvascular network engineering preparation techniques. Table 1 outlines the pros and cons of each technique for microvascular fabrication. To pre-vascularize engineered tissues and organs, different advanced techniques and biocompatible materials (including photo-responsive hydrogels, natural biomatrices and synthetic polymers) are combined to fabricate the microvasculature. Microfluidics is based mainly on self-assembly principles or microfabrication techniques to construct high-resolution vascular networks, which are particularly limited by minimum vascular size and geometry for microvascular fabrication. The use of photochemical reactions or laser energy techniques, such as photolithography, laser degradation, and 3D printing, have demonstrated advancements in terms of geometric complexity. Laser degradation is one of the methods used to fabricate vascular structures in target tissues with high resolution, guided by microscopic images. The ability of this method to create objects that adapt to external factors, such as temperature or humidity, and develop over time enables programmable 4D control of microvascular networks. Despite recent advances, aspects of the fabrication of capillary constructs in vitro remain challenging, including the endothelialization of capillary networks, the replication of complex microvascular spatial morphology, and the stabilization of capillary systems*.* To address these issues, efforts are needed to combine functional materials with sophisticated fabrication tools to directly control the morphology and mechanics of capillary structures.

Table 3 summarizes the advantages and disadvantages of the three cell types used for microvascular tissue engineering. Among the various endothelial cell subtypes, HUVECs have been predominantly used in most studies. However, HUVECs are derived from large veins so may not fully recapitulate the natural microvasculature such as small arteries and capillaries. Therefore, further studies are needed to culture and characterize suitable cell types (e.g. iPSCs).

The factors influencing angiogenesis are complex, with cells sensing physical and biological signals in the ECM and translating them into different cellular behaviors such as elongation, alignment, chemokine secretion, collagen production and remodeling. Innovative materials showing dynamic adjustability by integrating various influential aspects may hold promise in this area. However, numerous studies have concentrated on the impact of biological and mechanical elements on angiogenesis within the vasculature, while chemical factors that could affect cellular behavior, such as whether particular chemical bonds in the ECM influence the angiogenic behavior of cells, have not yet been explored. This presents an area for future investigation.

Vascular engineering has made significant progress. Engineered microvascular models mimic the complex physiological microenvironments present in living systems, facilitating the study of molecular mechanisms of physiological and pathological activities in tissues and organs. However, despite these revolutionary advances, there are still many challenges for future research. The capillary networks engineered do not precisely replicate the microvascular networks of native tissues. It remains a challenge to achieve hierarchical networks of microvascular networks, heterogeneity, and functionality. Engineered microvascular systems have limited clinical and industrial applications due to endothelial homeostasis, vascular perfusion, permeability, and constriction, which prevent their long-term maintenance in vitro. Additionally, due to the high cost of materials, machinery and equipment, it is difficult to produce them in large quantities on an industrial scale.

In the future, with developments and innovation in basic vascular biology, stem cell biology, biomedicine, materials science and other fields, we hope to comprehensively use functional materials, suitable cell types and complex microenvironmental features to construct high-throughput, high-resolution capillary networks with real complex spatial structures. The resulting capillary networks have a dual function. Firstly, they enable the simulation of physiological capillaries in vivo for in vitro studies. Secondly, they serve as pre-vascularized tissue-engineered constructs that can be implanted and perfused in vivo, providing tissues and organs with the necessary oxygen and nutrients.

## Data Availability

The authors can not provide any data and materials.

## Abbreviations

3D: Three-dimensional
ECs: Bioprinting, endothelial cells
ECM: Extracellular matrix
UV: Ultraviolet
PDMS: Polydimethylsiloxane
MSCs: Mesenchymal stem cells
BCAECs: Bovine carotid artery endothelial cells
HUVECs: Human umbilical vein endothelial cells
PF-127: Pluronic F127
GelMA: Gelatin methacrylate
MA: Methacrylate
hMSCs: Human mesenchymal stem cells
PEGDA: Poly (ethylene glycol) diacrylate
PEG: Polyethylene glycol
PLA: Polylactic acid
BMECs: Brain microvascular endothelial cells
HPMEC: Human pulmonary microvascular endothelial cells
hPSCs: Human pluripotent stem cells
hESCs: Human embryonic stem cells
hiPSCs: Human induced pluripotent stem cells
PDLGA: Poly(d-lactide-co-glycolide)
BM-hMSCs: Bone marrow-derived human mesenchymal stem cells
PcycloPrOx: Poly(2-cyclopropyl-2-oxazoline)
aFGF: Acidic fibroblast growth factor
bFGF: Basic fibroblast growth factor
2D: Two-dimensional
VEGF: Vascular Endothelial Growth Factor
HIF-1a: Hypoxia-inducible factor-1a
HMEC: Human microvascular endothelial cells
CaO_2_: Calcium peroxide
EF: Electric fields
VEGFR-2: Vascular Endothelial Growth Factor Receptor 2
ROS: Reactive oxygen species
